# Long-term efficacy and safety of paliperidone 6-month formulation: An open-label extension of a double-blind study in adult patients with schizophrenia

**DOI:** 10.1192/j.eurpsy.2023.345

**Published:** 2023-07-19

**Authors:** D. Najarian, I. Turkoz, S. Galderisi, H. F. Lamaison, P. Zalitacz, S. Aravind, U. Richarz

**Affiliations:** 1 Janssen Scientific Affairs, LLC; 2 Janssen Research & Development, LLC, Titusville, NJ, United States; 3 University of Campania “Luigi Vanvitelli”, Naples, Italy; 4 Department of Psychiatry, National University of La Plata (UNLP), Buenos Aires, Argentina; 5 Head of Psychiatric Unit, Gorlice Specialist Hospital, Gorlice, Poland; 6 Janssen Research & Development-Cilag, Gubelstrasse, Zug, Switzerland

## Abstract

**Introduction:**

Paliperidone palmitate 6-month (PP6M), administered twice-yearly, demonstrated non-inferiority to paliperidone palmitate 3-month (PP3M) in preventing relapse in patients with schizophrenia in a phase-3 randomized, double-blind (DB) global study.^1^ We report results of a 2-year single-arm, open-label extension (OLE) of this study (NCT04072575).

**Objectives:**

To assess long-term efficacy and safety of PP6M in patients with schizophrenia.

**Methods:**

Patients who completed DB study without relapse were enrolled and followed up every 3 months for up to 2 years. Patients received 4 PP6M injections (700/1000 mg eq.) at baseline, 6-month, 12-month, and 18-month visits. Efficacy endpoints included relapse rate, Positive and Negative Syndrome Scale (PANSS) total score, Personal and Social Performance (PSP) score, and Clinical Global Impression-Severity (CGI-S) scale change from baseline. Safety was assessed by treatment-emergent adverse events (TEAEs), physical examinations and laboratory tests.

**Results:**

Of 178 patients, 154 (86.5%) completed the study; mean age: 40.4 years; 70.8% were men. Mean duration of PP6M exposure was 682.1 days. Overall, 7/178 (3.9%) patients relapsed between 20 to 703 days after enrolment. Mean (SD) change from baseline to endpoint: PANSS total score, 0.7 (8.22); CGI-S, 0.0 (0.51); PSP Scale, 0.5 (7.47). Overall, 111/178 patients (62.4%) reported ≥1 TEAE; most common (>10%) TEAEs were headache (13.5%) and blood prolactin increased (10.7%). Total, 7/24 patients withdrew due to TEAEs, and 8/178 (4.5%) patients experienced serious TEAEs; no deaths were reported.

**Conclusions:**

Relapse rate with PP6M was very low (<4%). Clinical improvements in PANSS, CGI-S, and PSP scales demonstrated in DB study were maintained during this 2-year OLE study and no new safety concerns were identified.

**Reference:**

1. D. Najarian et al. *Int J Neuropsychopharmacol.* 2022 Mar 17;25(3):238-251.

**Disclosure of Interest:**

D. Najarian Shareolder of: Johnson & Johnson, Employee of: Janssen Research & Development, LLC, I. Turkoz Shareolder of: Johnson & Johnson, Employee of: Janssen Research & Development, LLC, S. Galderisi Consultant of: Janssen, Gedeon Richter-Recordati, Angelini, Speakers bureau of: Angelini, Gedeon Richter-Recordati, Janssen, Lundbeck, Sunovion, Recordati, H. Lamaison Grant / Research support from: Novartis, Eli Lilly, Lundbeck, Servier, AstraZeneca, Wyeth, Pfizer, Otsuka, Takeda, Sunovion, Roche, Janssen Pharmaceutical, Speakers bureau of: Servier, Abbot, Raymonds, Raffo, Temis Lostalo and Janssen Pharmaceutical, P. Zalitacz: None Declared, S. Aravind Shareolder of: Johnson & Johnson, Employee of: Advarra, Inc. USA, U. Richarz Shareolder of: Johnson & Johnson, Employee of: Janssen Research & Development-Cilag, Switzerland.

